# Optimizing the Nutritional Support of Adult Patients in the Setting of Cirrhosis

**DOI:** 10.3390/nu9101114

**Published:** 2017-10-13

**Authors:** Brandon J. Perumpail, Andrew A. Li, George Cholankeril, Radhika Kumari, Aijaz Ahmed

**Affiliations:** 1 Department of Medicine, Drexel University College of Medicine, Philadelphia, PA 19129, USA; bjp63@drexel.edu; 2 Division of Gastroenterology and Hepatology, Stanford University School of Medicine, Stanford, CA 94305, USA; andrewli@stanford.edu (A.A.L.); georgetc@stanford.edu (G.C.); rkumari@stanford.edu (R.K.)

**Keywords:** nutrition, cirrhosis, end-stage liver disease, Child-Turcotte-Pugh, malnutrition, dietary intervention, improved oral intake

## Abstract

Aim: The aim of this work is to develop a pragmatic approach in the assessment and management strategies of patients with cirrhosis in order to optimize the outcomes in this patient population. Method: A systematic review of literature was conducted through 8 July 2017 on the PubMed Database looking for key terms, such as malnutrition, nutrition, assessment, treatment, and cirrhosis. Articles and studies looking at associations between nutrition and cirrhosis were reviewed. Results: An assessment of malnutrition should be conducted in two stages: the first, to identify patients at risk for malnutrition based on the severity of liver disease, and the second, to perform a complete multidisciplinary nutritional evaluation of these patients. Optimal management of malnutrition should focus on meeting recommended daily goals for caloric intake and inclusion of various nutrients in the diet. The nutritional goals should be pursued by encouraging and increasing oral intake or using other measures, such as oral supplementation, enteral nutrition, or parenteral nutrition. Conclusions: Although these strategies to improve nutritional support have been well established, current literature on the topic is limited in scope. Further research should be implemented to test if this enhanced approach is effective.

## 1. Introduction

Malnutrition has become increasingly common in end-stage liver disease [1]. The prevalence of malnutrition has been reported in a significant proportion of patients with cirrhosis and ranges from 10% to 100%, contingent on severity of hepatic decompensation in the setting of cirrhosis [2,3]. However, even with this high occurrence, malnutrition is still under-diagnosed and ineffectively treated [4]. More specifically, many patients who are awaiting liver transplantation develop various nutritional deficiencies [1]. Malnutrition is a predictor of morbidity and mortality in patients with cirrhosis [3]. Malnutrition related to liver disease has been linked to a risk of infections, complications associated with surgery, poor candidacy for liver transplantation, and a prolonged length of stay in the hospital or intensive care unit [2,5]. Routine screening of patients with end-stage liver disease for malnutrition can facilitate prompt diagnosis leading to timely initiation of treatment and improved outcomes [6,7].

## 2. Malnutrition in End-Stage Liver Disease

In the presence of cirrhosis, malnutrition is diagnosed and defined as a deficiency in nutrients [8]. Under normal post-prandial conditions, ingested carbohydrates are metabolized by the liver and stored as glycogen. Subsequently, during the normal fasting period glycogen can be broken down (glycogenolysis) to glucose (gluconeogenesis) to maintain stable blood glucose levels (glucose homeostasis). Patients with cirrhosis have a compromised ability to store glycogen and blunted gluconeogenesis. Therefore, cirrhotic patients may enter a starvation state between dinner and breakfast, stimulating lipid oxidation, thus shifting from carbohydrate to fat as the main source of energy metabolism. A complex interaction of many factors are involved in increasing the risk of malnutrition in cirrhosis [9]. Poor calorie intake is a common contributor in these patients, which often stems from a lack of appetite or anorexia. The molecular mechanisms involved in malnutrition in the cirrhotic state are not fully understood and include zinc deficiency, early satiety resulting from an increased amount of leptin and tumor necrosis factor-alpha in the blood, etc. [8,9]. In addition, many patients may develop a poor appetite due to ascites or hepatic encephalopathy [10]. The resulting dietary restriction of salt may make food unpalatable, further escalating poor nutritional status [11]. Another cause of malnutrition is impaired absorption and digestion resulting from a host of factors, such as decreased bile-salt, bacterial overgrowth, and portal hypertension [8]. Finally, hypermetabolism leads to malnutrition through increased energy expenditure and is an independent predictor of both transplant-free and post-transplant survival [8,10,12]. This is mainly caused by changes in nutrient utilization due to the lack of available carbohydrates for energy, which places the body in a state of starvation, especially overnight [12]. Infection and ascites also contribute to the hypermetabolic state [10]. In the setting of cirrhosis, protein-energy malnutrition (PEM) is noted in up to two-thirds of affected individuals and correlates with Child-Turcotte-Pugh classification. PEM is associated with poor survival in this patient population. The contributing factors to PEM include hypermetabolism, poor caloric intake, malabsorption, increased intestinal permeability leading to protein loss, and decreased hepatic protein synthesis. In addition, PEM leads to muscle wasting manifested by a reduction in skeletal muscle volume and strength (secondary sarcopenia related to cirrhosis). Secondary sarcopenia associated with cirrhosis is an independent predictor of poor survival. Furthermore, certain disease-specific issues impacting the management of malnutrition must be individualized. For example, alcohol abuse associated with low socio-economic status and poor psycho-social support may pose a complex challenge in the management of malnutrition.

In order to fully address the issue of malnutrition in end-stage liver disease, improvement must be made in promptly diagnosing patients in need for nutritional support.

## 3. Improved Assessment of Malnutrition

**Figure 1:**
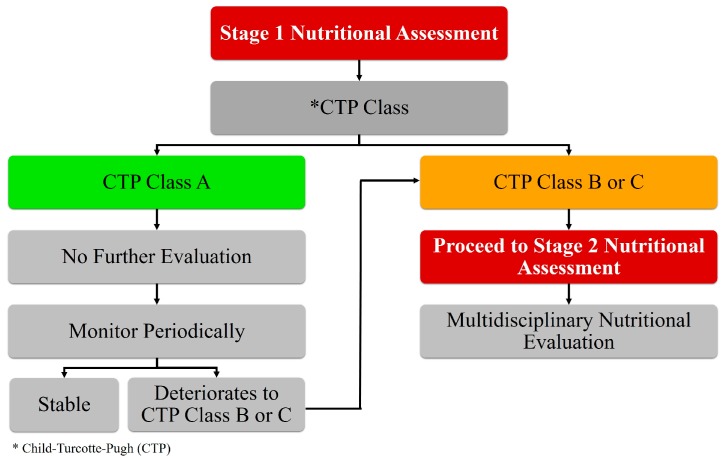
Two-stage approach to nutritional assessment in cirrhosis.

A systematic approach to screen for malnutrition in cirrhotic patients must be established to more directly address the growing problem of malnutrition. During the initial evaluation (stage 1 assessment), cirrhotic patients with immediate need for nutritional support must be identified. Subsequently, these selected patients must undergo a standardized evaluation (stage 2 assessment) leading to individualized patient care with a focus to optimize the nutritional status. This two-stage approach is summarized in Figure 1.

### 3.1. Stage 1 Assessment for Malnutrition: Define Immediate-Need Populations

The goal of a two-stage approach is to maximize the allocation of time and resources to those who need it the most to improve overall outcomes. Not every patient needs an immediate full evaluation (stage 2 assessment) for malnutrition; patients who are the most at risk should be targeted first and assessed. Malnutrition severity is strongly associated with the severity of hepatic decompensation (liver failure) in the setting of cirrhosis [3,13]. A Chinese study evaluating malnutrition in patients with chronic liver disease found that patients with the highest rate of nutritional risk were also those with the greatest degree of liver failure (patients in Child-Turcotte-Pugh (CTP) classes B and C) [13]. The two-stage approach uses the association between malnutrition and severity of liver failure to identify the patient population in immediate need of nutritional assessment using the CTP scoring system. This classification, created by Child and Turcotte in 1964 and later adjusted in 1973 by Pugh, is a commonly-used tool for measuring the severity of liver failure in cirrhotic patients and to estimate the risk of surgical procedures [14,15,16]. The CTP system scores 1–3 points, with one being the most normal and three being the most abnormal, in five different categories (albumin, bilirubin, prothrombin prolongation time, and hepatic encephalopathy) to grade patients into three classes: A (5–6 points), B (7–9 points), and C (10–15 points), with classes B and C being the most severe [17]. This constitutes the ‘stage 1 assessment’ and identifies patients with cirrhosis at the highest risk of malnutrition, namely CTP classes B and C [13]. Another study evaluated of cirrhotic patients for malnutrition categorized by the three CTP classes and noted malnourishment in more than half the patients in the CTP class B and C groups, 77.3% and 94.4% respectively, while less than half of the patients in CTP class A developed malnutrition [3]. In a study that compared the Subjective Global Assessment (SGA) of patients’ nutrition with their CPT class, only patients in class B and C were indicated to have levels of malnutrition ranging from moderate to severe [11]. Thus, cirrhotic patients with CTP class B and C have been shown to be the most likely to develop malnutrition and require comprehensive ‘stage 2 assessment’ for poor nutritional status.

### 3.2. Stage 2 Assessment for Malnutrition: Multidisciplinary Nutritional Assessment

**Figure 2:**
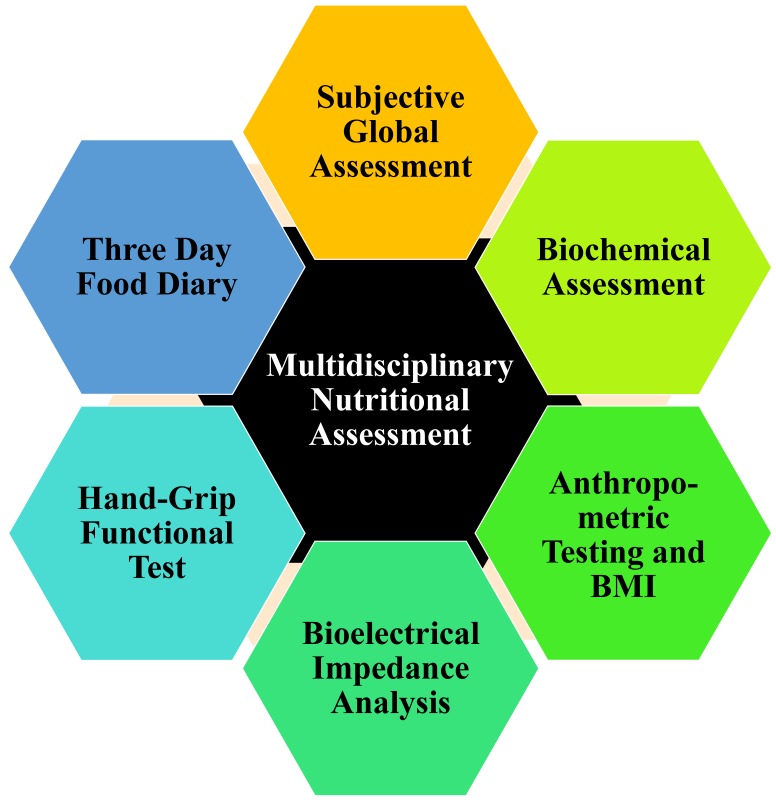
Multidisciplinary nutritional assessment.

A more thorough and comprehensive evaluation of the nutritional status can be achieved and a focused individualized approach can be pursued. This not only allows for proper apportionment of resources and time, but also provides a more targeted approach in which patients can be prioritized based on the severity of malnutrition. A study evaluating protein depletion in patients with cirrhosis concluded that the utilization of multiple nutritional assessment tools were crucial in making an accurate assessment of severity of malnutrition [18]. End-stage liver disease is a confounding factor that affects the results of multiple nutritional assessment tools and, thus, there has to be confirmation across many techniques to diagnose malnutrition [5]. For example, weight and albumin concentrations could be unreliable measurements alone due to other complications from cirrhosis, such as ascites or low protein synthesis, respectively [10,18,19]. Other cross-sectional studies looking at nutritional assessment techniques found that the use of a variety of techniques, such as the SGA, biochemical tests, and anthropometry, lead to a better understanding of nutritional status [2,20,21,22]. A variety of tests should be used to evaluate different aspects and severity of nutrition rather than any single nutritional screening tool, thus creating a multidisciplinary approach shown in Figure 2 [1,19].

A standard nutritional evaluation often used in hospitals is the SGA [23]. It is an attractive test due to its accuracy while also being cheap, non-invasive, and simple to execute [23]. Multiple studies compare the SGA results with other nutritional measurement tools and have found it to provide consistent results, validating the SGA’s precision and specificity [2,22,24]. It was also studied in the context of liver transplantation and was shown to predict complications during transplantation, as well as post-operative outcomes [5]. The SGA is a questionnaire with two main components, history and physical examination [25]. The history portion asks questions regarding previous weight changes over time, recent alterations of food intake, recent functionality, and symptoms affecting intake [25]. The physical examination measures fat loss, muscle wasting, and the presence of edema and ascites [25]. A score is calculated based on the evaluation and the patient is assigned to one of three stages—Stage A, well nourished; Stage B, moderately malnourished; or Stage C, severely malnourished, with each stage having a list of features characterizing the multiple categories assessed [25].

A biochemical assessment for markers of malnutrition is commonly used for monitoring nutrition [1]. The test should include many factors, such as hemoglobin, albumin, white blood cell count, retinol-binding protein, transferrin, liver function exams, glucose, cholesterol, urea nitrogen, C-reactive protein, pre-albumin, nitrogen balance, creatinine, sodium, magnesium, zinc, potassium, and others [19,20]. Serum albumin is a common tool used to measure nutritional status and can help categorize the level of malnutrition [20]. However, one issue is that albumin also declines due to hepatic damage, making it an unreliable marker [19,26]. Pre-albumin and transferrin are impacted by malnutrition and hepatic damage similarly, but respond faster and, therefore, could also be used as early indications of malnutrition [26]. Retinol binding protein (RBP) relies on the presence of other nutrients, such as vitamin A and zinc, in order to carry out its function. Thus, micronutrient deficiency due to malnutrition would affect the blood levels of RBP as well [27]. Nitrogen balance is a good measure of dietary protein intake and protein metabolism [28]. Deficiency occurs when protein metabolism is greater than protein intake, as indicated by a negative nitrogen balance [28].

Furthermore, anthropometric testing and body mass index (BMI) are necessary tools when assessing nutritional body composition [29]. Anthropometric testing measures height, weight, mid-arm circumference, mid-arm muscle circumference, triceps skin fold thickness, and biceps skin fold thickness [20,21]. BMI and anthropometric measures can be used to assess skeletal muscle mass and adipose deposit levels [30,31,32]. A recent study found that thigh muscle thickness when used with BMI can be an important tool in identifying sarcopenia and malnutrition [31]. A potential problem with BMI is that edema and ascites could increase the measured weight and make BMI inaccurate; however, dry BMI can be measured by using corrective factors for dry weight that account for the level of edema. Five, ten, or fifteen percent are subtracted from the measured weight for mild, moderate, or severe ascites, and an extra 5% would be subtracted if edema is present [30,31]. Another way to account for ascites is to use the established BMI standards for ascites where BMI below or equal to 22 with no ascites, a BMI of 23 kg/m [2] with mild ascites, or a BMI of 25 kg/m [2] with tension ascites is considered a state of malnutrition [33]. Another useful tool to measure body composition is bioelectrical impedance analysis (BIA) [33,34,35]. BIA is swift, simple, and non-invasive, which makes it an attractive new technique that is gaining interest [35,36,37]. It conducts an electric current through the body and calculates its electric impedance, which is made up of two components—resistance, which measures body water, and reactance, which measures body cell mass (BCM). Based on this information, measurements about body composition can be estimated, such as BCM, BCM%, extracellular mass, mass that is not from body fat, body fat mass, phase angle, and total body water [34,37,38]. This information can be utilized to understand various aspects of body composition and determine how they change over time with intervention [33]. For example, BIA can calculate body water, which could be indicative of edema or ascites [33]. Furthermore, recent research has found that phase angle is a better predictor of nutritional status in cirrhosis than anthropomethric evaluation tools and can even be used for early diagnosis of malnutrition [35].

Functional testing is another important evaluation when assessing nutrition; this allows for a means of testing the strength and ability of the skeletal muscle [2]. A longitudinal study found that although muscle strength and muscle mass both deteriorate with malnutrition, the former declines at a much quicker rate [39]. Additionally, muscle strength can decrease without a loss in muscle mass [39]. Hand-grip strength is a tool used to assess muscle strength and has been validated as an independent method of identifying malnutrition and muscle capability [40]. Moreover, a comparative study found that when tested against other nutritional assessment tools, such as SGA, BMI, and anthropometric measurements, the hand-grip strength test had the highest accuracy for detecting the presence of nutritional compromise in the context of liver disease [41]. Another study found that not only does hand-grip strength correlate with nutritional level, it also predicts hospital outcomes, such as post-operative complications, length of hospitalization, risk of re-hospitalization, and physical ability.

The final portion of the assessment is an evaluation of dietary intake [2]. The best method to record this is through a three-day food diary with specific instructions given to the patient on how to complete the diary so that accurate information on food consumption can be documented [2]. Many studies have validated the three-day food diary as an effective measure of dietary intake and it has been shown to be more accurate than other methods such as the food frequency questionnaire and 24-h recall [42,43]. The food diary helps physicians and dieticians understand the different food habits, dietary choices, and overall consumption levels (calories and protein content) of the patient over time so that they can assess nutrition in an individualized manner [44]. The use of multiple tools and tests to evaluate the patient’s nutritional status creates a well-rounded image of the patient’s health so that an assessment of malnutrition are both thorough and specific. An initial assessment stage that filters patients based on severity of hepatic decompensation in the setting of cirrhosis allows only the malnourished or most at-risk for malnutrition to proceed to the next stage. Furthermore, this individualized approach results in pragmatic utilization and allocation of restricted resources.

## 4. Treatment Options for Malnourished Patients

Malnutrition in the setting of cirrhosis has been noted as a reliable indicator of quality of life and key predictor of inpatient outcomes (morbidity and mortality) [3]. Studies have indicated that these outcomes can be improved by optimizing the nutritional status of a patient with cirrhosis [7]. Unfortunately, malnutrition remains an alarmingly prevalent problem, especially in patients with cirrhosis [3,45]. Therefore, it is very important to develop an objective and standardized initial and follow-up evaluation pathway/management protocol to assess the nutritional status of patients with cirrhosis. In particular, patients with hepatic decompensation/liver failure awaiting liver transplantation require an ongoing evaluation of their nutritional status in the era of donor organ shortage and longer waiting times on transplant waitlists.

### 4.1. Proper Dietary Recommendations and Education

In patients with cirrhosis, the recommendation for different macromolecules has changed over time and health care practitioners need to be aware of these changes so that patients are properly managed [12]. Gastroenterology and hepatology fellowships do not always offer a comprehensive training in nutritional assessment and treatment; therefore, physicians may not always know the best methods available for diagnosing or treating patients who face malnourishment [9]. The most important aspect of malnourishment management is ensuring that the patient’s rehabilitative diet has the correct amount of each essential nutrient or macromolecule according to the current regulations [12]. Furthermore, diet plays a substantial role in cirrhosis and the related severity of hepatic decompensation/liver failure. Inadequate diets have been associated with further progression of liver disease and increased risk of cirrhosis. However, proper diet alterations have been noted to not only prevent disease progression, but also to reduce the severity of liver failure [46,47]. The recommendation for malnourished patients with cirrhosis is 35–40 kcal/kg/day to promote anabolism [33,48]. Protein deficiency is a significant problem in malnutrition which can be addressed through an intake of 1.2–1.5 g/kg/day [48,49]. Furthermore, hepatic damage causes an increase in aromatic amino acids (AAA) and decreases branched-chain amino acids (BCAA) which can lead to hepatic encephalopathy and other neurological complications [29]. Studies have found that increasing the BCAA to AAA ratio through improved dietary BCAA intake has led to normalization and increased survival [12,50]. The guideline for carbohydrates is 50–70% of daily calories [51]. However, simple sugars, especially fructose, should be avoided as much as possible [6]. The recommendation for lipids is 10–20% of calories with the majority being monounsaturated and polyunsaturated fatty acids [6,11]. Special considerations have to be taken for hepatic encephalopathy and ascites [33]. In hepatic encephalopathy, there should be an increased emphasis on BCAAs and fiber with decreased ammonia [33]. Previous recommendations for hepatic encephalopathy included decreased protein intake, but more recent data have shown that this practice is outdated and incorrect [6,9,10]. Patients with ascites should be on a low-sodium diet (less than or equal to 2 g/day) and should also have water restriction when edema is present or if hyponatremia occurs [33]. Ensuring patients meet these requirements, organized in Table 1, is the first step in optimizing nutritional support in patients with end-stage liver disease. In patients with cirrhosis associated with nonalcoholic steatohepatitis caloric restriction, but not protein restriction, can be recommended. Furthermore, patients with fluid retention must be educated to restrict their sodium intake to less than 2 g per day and fluid intake of 2 L per day.

### 4.2. Techniques to Promote Oral Intake

**Figure 3:**
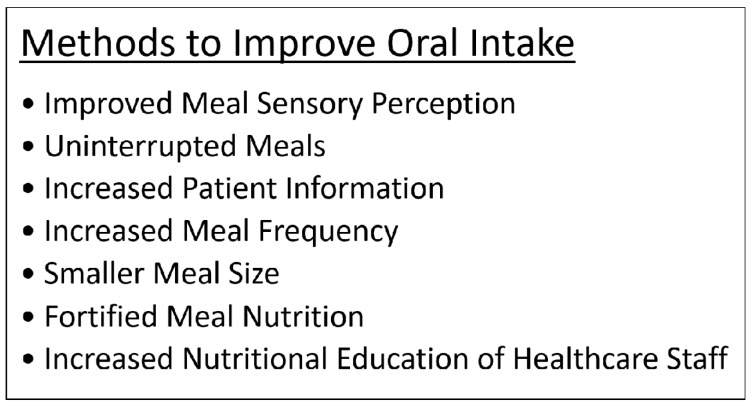
Methods to improve oral intake.

In general, adequate oral intake has been a difficult task for many patients facing malnutrition related to cirrhosis and other chronic ailments [52]. Patients with malnutrition are unable to maintain their daily caloric targets and requirements for nutrients despite institution of reinforced diet plans [52]. Many studies have reported that patients with malnutrition have significant amounts of calories wasted with each partially-ingested meal [53,54]. One study questioned patients after meals at the hospital in an attempt to understand the reasoning behind incomplete meal intake and suspected that meal sensory perception affected eating capability despite hunger [55]. Patients responded regarding various aspects of their meal experience (appearance, aroma, taste, texture, temperature, and food variety) and requested that more attention be paid to provide a more individualized eating experience [55]. These factors should be continuously re-evaluated to reflect the preferences of the patients as their appetite changes based on the severity of hepatic decompensation [55]. In addition to this study, others have shown that meal presentation and appearance impacts the desire to eat; patients ate more from a neatly-arranged plate rather than a disorganized one [54,55]. Additionally, overwhelming odors from food discouraged eating [55]. For example, when feeling nauseous, patients desired meals that had neutral tastes [55]. Often softer foods that were easier to ingest were associated with increased intake [55,56]. A hot meal versus a cold meal was more likely to be ingested [55]. Another study assessing issues with oral intake showed that interruptions during meal times by nurses, physicians, or tests led to missed meals that were never served later; patients were also unaware of the options available for extra snacks between meals [53]. This suggests that patients should have scheduled meal times that are protected and nursing staff should ensure that the patients eat during the dedicated meal period without interruption, plus improving patient awareness regarding additional accessible snacks can increase caloric intake [53]. Studies to improve intake noted that smaller meals, increased meal frequency, and fortified nutrition within these meals were effective in instituting increased nutritional support [57]. Furthermore, studies in the setting of cirrhosis found that 4–6 meals high in carbohydrates with an evening snack fortified with BCAAs prevented hypoglycemia and led to increased nutrition due to reduced catabolism overnight [8]. Another important factor found in studies was the level of nursing education [58]. Poulsen and colleagues [58] noticed increased nutrition and oral intake in patients when nurses were given a short class on nutritional care to provide individualized nutritional support. Figure 3 summarizes the various interventions that can be used to promote oral intake and improve nutritional status of patients with cirrhosis.

### 4.3. Alternative Feeding Methods

Although oral intake of food is the preferred route of nutrition, factors, such as patient weakness or inability to eat, may necessitate pursuit of alternative methods for nutrient delivery [10,59]. Oral supplements should be used in an individualized approach to fulfill any specific nutrient deficiencies a patient may have [10,59]. A clinical trial noted that patients given oral supplements between meals met the recommended nutritional intakes [59]. Other options for patients unable to handle oral intake are enteral nutrition through a nasogastric tube and, if necessary, parenteral nutrition [10]. Both of these have been shown to be effective in various studies assessing malnutrition [10,60]. Complications of cirrhosis and end-stage liver disease associated with gut microbiota dysfunction, such as hepatic encephalopathy, are effectively managed with prebiotics, probiotics and synbiotics. Optimal management of hepatic encephalopathy and favorable gut microbiota may lead to improved nutritional status in patients with cirrhosis. Herbal remedies and supplements should be avoided with cirrhosis due to increased risk of hepatotoxicity with marginal hepatic reserve.

## 5. Conclusions

Malnutrition is a growing problem, especially in cirrhotic patients with end-stage liver disease. The enhancement of methods to assess and treat malnutrition is the key to optimizing patient outcomes. Assessment of malnutrition should be done in two stages, the first to identify patients at risk for malnutrition based on cirrhosis and the second to run a complete multidisciplinary nutritional evaluation on these patients. Treatments for malnutrition should make sure patients reach the recommended daily caloric and nutrient goals by increasing oral intake or by using other measures, such as oral supplementation, enteral nutrition, or parenteral nutrition. Further prospective data are needed from dedicated studies to optimize the nutritional status and outcomes in patients with cirrhosis improved through proper nutritional support of patients with advanced liver disease [7]. Therefore, standardized protocols to screen for malnutrition associated with cirrhosis and timely intervention with nutritional support is a pivotal step in reducing the risk of morbidity and mortality.

## Figures and Tables

**Table 1:** Nutritional recommendations for malnutrition in cirrhosis.

**Nutritional Recommendations for Malnutrition in Cirrhosis**	
Daily Calories	35–40 kcal/kg/day
Proteins	1.2–1.5 g/kg/day with increased BCAAs
Carbohydrates	50–70% of daily calories with decreased simple sugars—especially fructose
Lipids	10–20% of daily calories with increased MUFAs and PUFAs
**Special Considerations**	
Hepatic Encephalopathy	Maintain protein intake, increase BCAAs and decreased ammonia intake
Ascites	Low-sodium diet (≤2 g/day) and water restriction when necessary

## References

[1] Lim H.S., Kim H.C., Park Y.H., Kim S.K. (2015). Evaluation of Malnutrition Risk after Liver Transplantation Using the Nutritional Screening Tools. Clin. Nutr. Res..

[2] Marr K.J., Shaheen A.A., Lam L., Stapleton M., Burak K., Raman M. (2017). Nutritional status and the performance of multiple bedside tools for nutrition assessment among patients waiting for liver transplantation: A Canadian experience. Clin. Nutr. ESPEN.

[3] Maharshi S., Sharma B.C., Srivastava S. (2015). Malnutrition in cirrhosis increases morbidity and mortality. J. Gastroenterol. Hepatol..

[4] Cheung K., Lee S.S., Raman M. (2012). Prevalence and mechanisms of malnutrition in patients with advanced liver disease, and nutrition management strategies. Clin. Gastroenterol. Hepatol..

[5] Stephenson G.R., Moretti E.W., El-Moalem H., Clavien P.A., Tuttle-Newhall J.E. (2001). Malnutrition in liver transplant patients: Preoperative subjective global assessment is predictive of outcome after liver transplantation. Transplantation.

[6] McClain C.J. (2016). Nutrition in patients with cirrhosis. Gastroenterol. Hepatol..

[7] Alberda C., Gramlich L., Jones N., Jeejeebhoy K., Day A.G., Dhaliwal R., Heyland D.K. (2009). The relationship between nutritional intake and clinical outcomes in critically ill patients: Results of an international multicenter observational study. Intensive Care Med..

[8] Ye Q., Yin W., Zhang L., Xiao H., Qi Y., Liu S., Qian B., Wang F., Han T. (2017). The value of grip test, lysophosphatidlycholines, glycerophosphocholine, ornithine, glucuronic acid decrement in assessment of nutritional and metabolic characteristics in hepatitis B cirrhosis. PLoS ONE.

[9] Patton H.M. (2012). Nutritional assessment of patients with chronic liver disease. Gastroenterol. Hepatol..

[10] Patel J.J., McClain C.J., Sarav M., Hamilton-Reeves J., Hurt R.T. (2017). Protein requirements for critically Ill patients with renal and liver failure. Nutr. Clin. Pract..

[11] Teiusanu A., Andrei M., Arbanas T., Nicolaie T., Diculescu M. (2012). Nutritional status in cirrhotic patients. Maedica.

[12] Eghtesad S. (2013). Malnutrition in liver cirrhosis: The influence of protein and sodium. Middle East J. Dig. Dis..

[13] Shi S., Han J., Yan M., Wang K., Yu H., Meng Q. (2014). Nutritional risk assessment in patients with chronic liver disease. Zhonghua Gan Zang Bing Za Zhi.

[14] Child C.G., Turcotte J.G. (1964). Surgery and portal hypertension. Major Probl. Clin. Surg..

[15] Pugh R.N., Murray-Lyon I.M., Dawson J.L., Pietroni M.C., Williams R. (1973). Transection of the oesophagus for bleeding oesophageal varices. Br. J. Surg..

[16] Zhou C., Hou C., Cheng D., Tang W., Lv W. (2015). Predictive accuracy comparison of MELD and Child-Turcotte-Pugh scores for survival in patients underwent TIPS placement: A systematic meta-analytic review. Int. J. Clin. Exp. Med..

[17] Raszeja-Wyszomirska J., Wasilewicz M.P., Wunsch E., Szymanik B., Jarosz K., Wójcicki M., Milkiewicz P. (2009). Assessment of a modified Child-Pugh-Turcotte score to predict early mortality after liver transplantation. Transplant. Proc..

[18] Prijatmoko D., Strauss B.J., Lambert J.R., Sievert W., Stroud D.B., Wahlqvist M.L., Katz B., Colman J., Jones P., Korman M.G. (1993). Early detection of protein depletion in alcoholic cirrhosis: Role of body composition analysis. Gastroenterology.

[19] Bharadwaj S., Ginoya S., Tandon P., Gohel T.D., Guirguis J., Vallabh H., Jevenn A., Hanouneh I. (2016). Malnutrition: Laboratory markers vs nutritional assessment. Gastroenterol. Rep..

[20] Tai M.L., Goh K.L., Mohd-Taib S.H., Rampal S., Mahadeva S. (2010). Anthropometric, biochemical and clinical assessment of malnutrition in Malaysian patients with advanced cirrhosis. Nutr. J..

[21] Nunes F.F., Bassani L., Fernandes S.A., Deutrich M.E., Pivatto B.C., Marroni C.A. (2016). Food consumption of cirrhotic patients, comparison with the nutritional status and disease staging. Arq. Gastroenterol..

[22] Filipovic B.F., Gajic M., Milinic N., Milovanović B., Filipović B.R., Cvetković M., Sibalić N. (2010). Comparison of two nutritional assessment methods in gastroenterology patients. World J. Gastroenterol..

[23] Da Silva Fink J., Daniel de Mello P., Daniel de Mello E. (2015). Subjective global assessment of nutritional status—A systematic review of the literature. Clin. Nutr..

[24] Mourao F., Amado D., Ravasco P., Vidal P.M., Camilo M.E. (2004). Nutritional risk and status assessment in surgical patients: A challenge amidst plenty. Nutr. Hosp..

[25] Detsky A.S., McLaughlin J.R., Baker J.P., Johnston N., Whittaker S., Mendelson R.A., Jeejeebhoy K.N. (1987). What is subjective global assessment of nutritional status?. JPEN J. Parenter. Enteral Nutr..

[26] Fuhrman M.P., Charney P., Mueller C.M. (2004). Hepatic proteins and nutrition assessment. J. Am. Diet. Assoc..

[27] Chaves G.V., Peres W.A., Goncalves J.C., Ramalho A. (2015). Vitamin A and retinol-binding protein deficiency among chronic liver disease patients. Nutrition.

[28] Rand W.M., Pellett P.L., Young V.R. (2003). Meta-analysis of nitrogen balance studies for estimating protein requirements in healthy adults. Am. J. Clin. Nutr..

[29] Johnson T.M., Overgard E.B., Cohen A.E., DiBaise J.K. (2013). Nutrition assessment and management in advanced liver disease. Nutr. Clin. Pract..

[30] Tandon P., Ney M., Irwin I., Ma M.M., Gramlich L., Bain V.G., Esfandiari N., Baracos V., Montano-Loza A.J., Myers R.P. (2012). Severe muscle depletion in patients on the liver transplant wait list: Its prevalence and independent prognostic value. Liver Transpl..

[31] Tandon P., Low G., Mourtzakis M., Zenith L., Myers R.P., Abraldes J.G., Shaheen A.A., Qamar H., Mansoor N., Carbonneau M. (2016). A model to identify sarcopenia in patients with cirrhosis. Clin. Gastroenterol. Hepatol..

[32] Ponziani F.R., Gasbarrini A. (2017). Sarcopenia in patients with advanced liver disease. Curr. Protein Pept. Sci..

[33] Moctezuma-Velazquez C., Garcia-Juarez I., Soto-Solis R., Hernandez-Cortes J., Torre A. (2013). Nutritional assessment and treatment of patients with liver cirrhosis. Nutrition.

[34] Verney J., Metz L., Chaplais E., Cardenoux C., Pereira B., Thivel D. (2016). Bioelectrical impedance is an accurate method to assess body composition in obese but not severely obese adolescents. Nutr. Res..

[35] Fernandes S.A., de Mattos A.A., Tovo C.V., Marroni C.A. (2016). Nutritional evaluation in cirrhosis: Emphasis on the phase angle. World J. Hepatol..

[36] Dehghan M., Merchant A.T. (2008). Is bioelectrical impedance accurate for use in large epidemiological studies?. Nutr. J..

[37] Walter-Kroker A., Kroker A., Mattiucci-Guehlke M., Glaab T. (2011). A practical guide to bioelectrical impedance analysis using the example of chronic obstructive pulmonary disease. Nutr. J..

[38] Lee Y., Kwon O., Shin C.S., Lee S.M. (2015). Use of bioelectrical impedance analysis for the assessment of nutritional status in critically ill patients. Clin. Nutr. Res..

[39] Goodpaster B.H., Park S.W., Harris T.B., Kritchevsky S.B., Nevitt M., Schwartz A.V., Simonsick E.M., Tylavsky F.A., Visser M., Newman A.B. (2006). The loss of skeletal muscle strength, mass, and quality in older adults: The health, aging and body composition study. J. Gerontol. A Biol. Sci. Med. Sci..

[40] Flood A., Chung A., Parker H., Kearns V., O’Sullivan T.A. (2014). The use of hand grip strength as a predictor of nutrition status in hospital patients. Clin. Nutr..

[41] Sharma P., Rauf A., Matin A., Agarwal R., Tyagi P., Arora A. (2017). Handgrip strength as an important bed side tool to assess malnutrition in patient with liver disease. J. Clin. Exp. Hepatol..

[42] Crawford P.B., Obarzanek E., Morrison J., Sabry Z.I. (1994). Comparative advantage of 3-day food records over 24-h recall and 5-day food frequency validated by observation of 9- and 10-year-old girls. J. Am. Diet. Assoc..

[43] Yang Y.J., Kim M.K., Hwang S.H., Ahn Y., Shim J.E., Kim D.H. (2010). Relative validities of 3-day food records and the food frequency questionnaire. Nutr. Res. Pract..

[44] Ortega R.M., Perez-Rodrigo C., Lopez-Sobaler A.M. (2015). Dietary assessment methods: Dietary records. Nutr. Hosp..

[45] Rojas-Loureiro G., Servin-Caamano A., Perez-Reyes E., Servin-Abad L., Higuera-de la Tijera F. (2017). Malnutrition negatively impacts the quality of life of patients with cirrhosis: An observational study. World J. Hepatol..

[46] Pimentel C.F., Lai M. (2016). Nutrition interventions for chronic liver diseases and nonalcoholic fatty liver disease. Med. Clin. N. Am..

[47] Kim W. (2017). Treatment options in non-alcoholic fatty liver disease. Korean J. Gastroenterol..

[48] Chao A., Waitzberg D., de Jesus R.P., Bueno A.A., Kha V., Allen K., Kappus M., Medici V. (2016). Malnutrition and nutritional support in alcoholic liver Disease: A review. Curr. Gastroenterol. Rep..

[49] Putadechakum S., Klangjareonchai T., Soponsaritsuk A., Roongpisuthipong C. (2012). Nutritional status assessment in cirrhotic patients after protein supplementation. ISRN Gastroenterol..

[50] Hanai T., Shiraki M., Nishimura K., Ohnishi S., Imai K., Suetsugu A., Takai K., Shimizu M., Moriwaki H. (2015). Sarcopenia impairs prognosis of patients with liver cirrhosis. Nutrition.

[51] Anastácio L.R., Davisson Correia M.I.T. (2016). Nutrition therapy: Integral part of liver transplant care. World J. Gastroenterol..

[52] Thibault R., Chikhi M., Clerc A., Darmon P., Chopard P., Genton L., Kossovsky M.P., Pichard C. (2011). Assessment of food intake in hospitalised patients: A 10-year comparative study of a prospective hospital survey. Clin. Nutr..

[53] Van Bokhorst-de van der Schueren M.A., Roosemalen M.M., Weijs P.J., Langius J.A. (2012). High waste contributes to low food intake in hospitalized patients. Nutr. Clin. Pract..

[54] Navarro D.A., Boaz M., Krause I., Elis A., Chernov K., Giabra M., Levy M., Giboreau A., Kosak S., Mouhieddine M. (2016). Improved meal presentation increases food intake and decreases readmission rate in hospitalized patients. Clin. Nutr..

[55] Sorensen J., Holm L., Frost M.B., Kondrup J. (2012). Food for patients at nutritional risk: A model of food sensory quality to promote intake. Clin. Nutr..

[56] Forde C.G., van Kuijk N., Thaler T., de Graaf C., Martin N. (2013). Texture and savoury taste influences on food intake in a realistic hot lunch time meal. Appetite.

[57] Lorefalt B., Wissing U., Unosson M. (2005). Smaller but energy and protein-enriched meals improve energy and nutrient intakes in elderly patients. J. Nutr. Health Aging.

[58] Poulsen I., Vendel Petersen H., Rahm Hallberg I., Schroll M. (2007). Lack of nutritional and functional effects of nutritional supervision by nurses: A quasi-experimental study in geriatric patients. Scand. J. Food Nutr..

[59] Campbell K.L., Webb L., Vivanti A., Varghese P., Ferguson M. (2013). Comparison of three interventions in the treatment of malnutrition in hospitalised older adults: A clinical trial. Nutr. Diet..

[60] Kozeniecki M., Fritzshall R. (2015). Enteral nutrition for adults in the hospital setting. Nutr. Clin. Pract..

